# Supplementary material for: Uncovering mitotic ultrastructure in the native hair follicle using volume electron microscopy
Source: J Cell Sci. 2026 Feb 27;139(4):jcs264198. doi: 10.1242/jcs.264198 (PMC12967144; doi:10.1242/jcs.264198)
Supplement: Supplementary information [file joces-139-264198-s1.pdf]

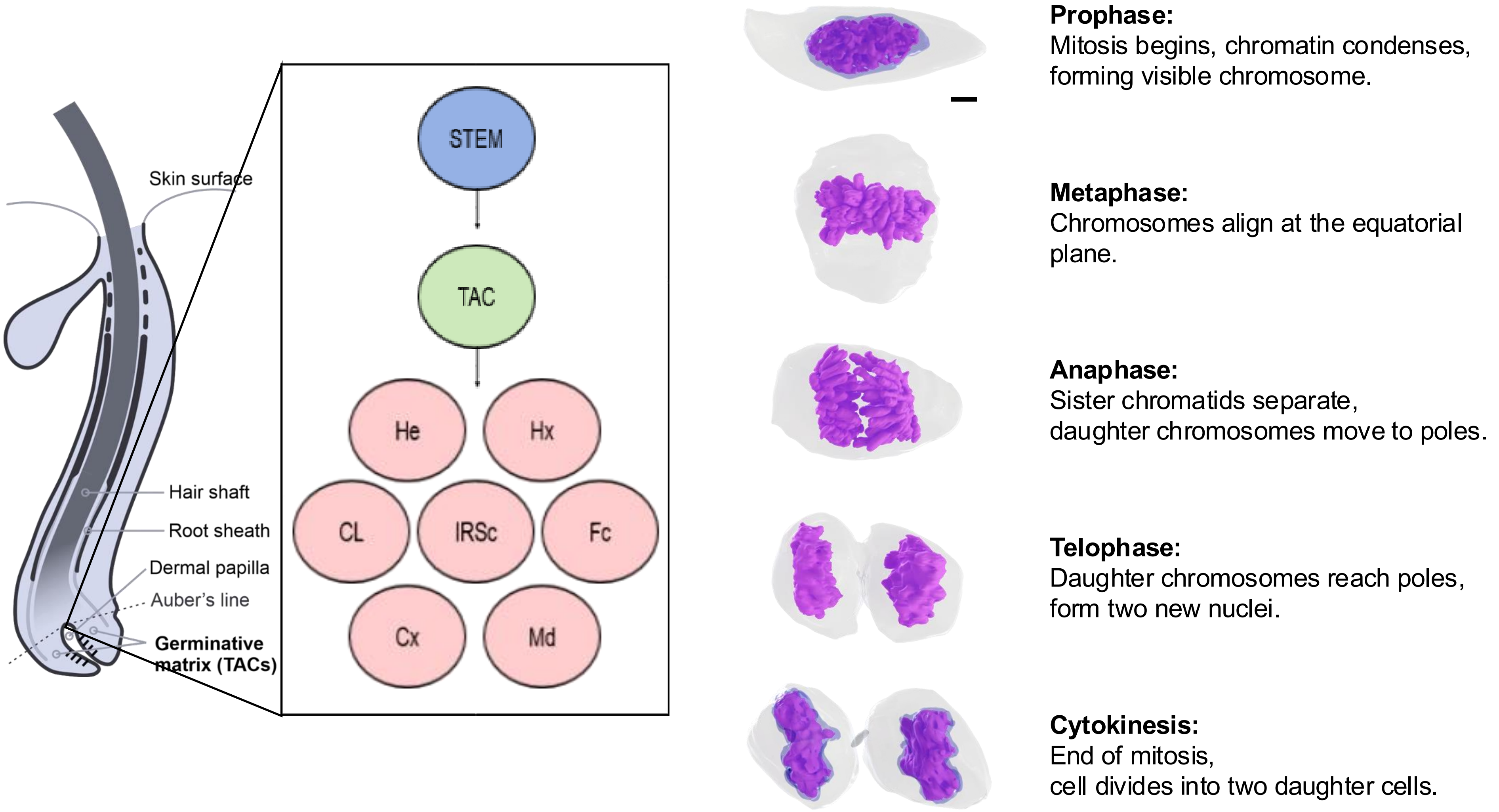

Fig. S1. ***Schematic of ovine hair follicle (HF) and* transit amplifying cell (TAC*) pathway.*** (**A**) Graphical representation of the HF, cell progression from STEM to terminally differentiated cells, featuring a reconstructed mitotic structures to illustrate the distinct phases: cell membrane (grey), nucleus (blue), and chromosomes (lilac) . This schematic provides a visual overview of the TAC pathway within the ovine HF structure, depicting the progression from Stem cells to TAC into seven ovine-specific matured cell types: CL (Cuticle layer), He (Henle's layer), Hx (Huxley's layer), IRSc (Inner Root Sheath cuticle), Fc (Fibrous cortex), Cx (Cortical cells), and MD (Medulla). A star indicates the point of transition where the mitotic cells are analysed. This encompasses the full mitotic stages from prophase to cytokinesis, illustrated as a 3D reconstructions derived from Serial Block-Face Scanning Electron Microscopy.
Scale bar to 2 µm.

Journal of Cell Science • Supplementary information

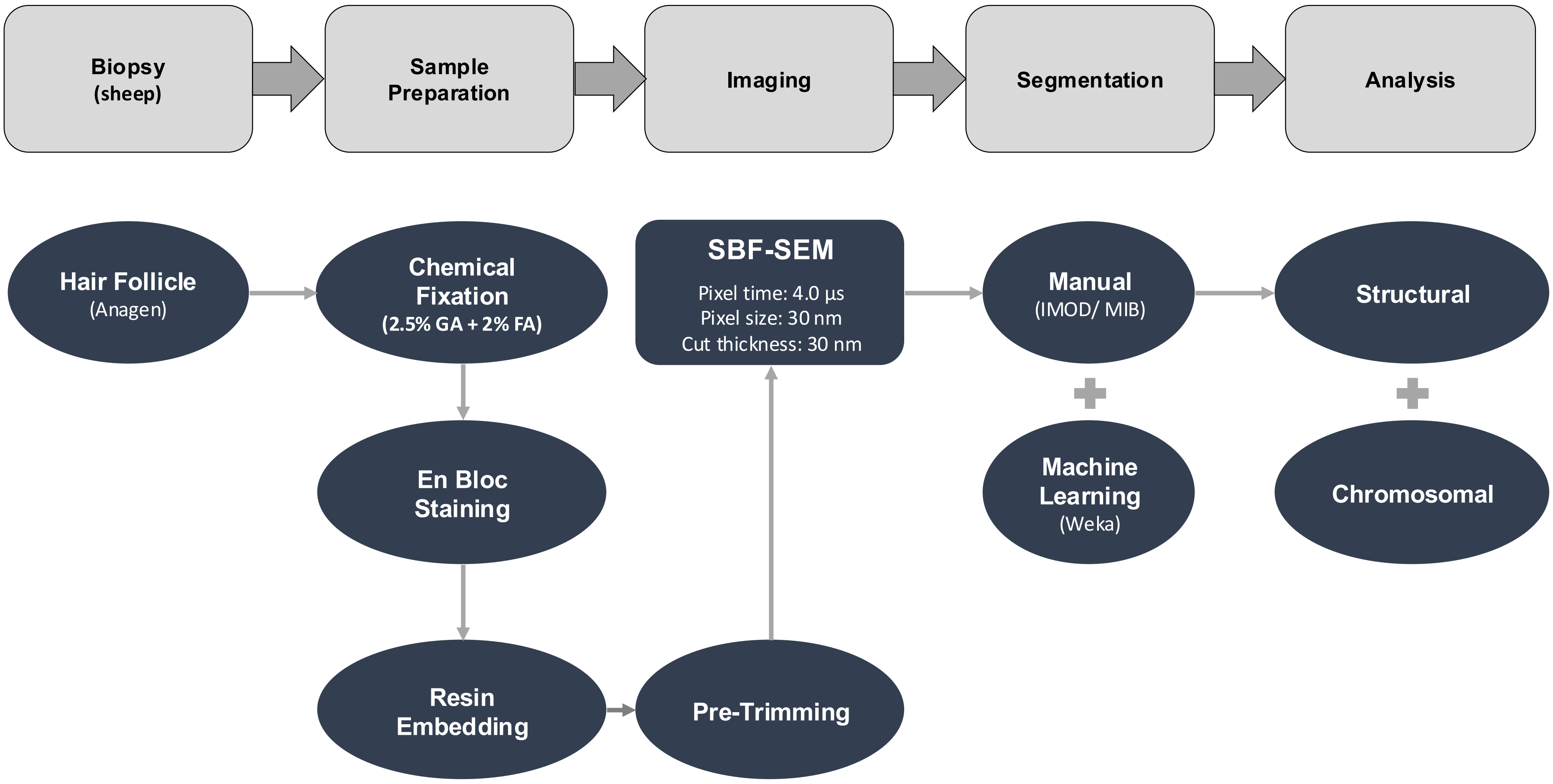

Fig. S2. ***Methodology overview for processing ultrastructural analysis of ovine hair follicle sample.*** Main steps outlined from biopsy to sample preparation, imaging, segmentation, and analysis. For a more detailed procedure, refer to the methodology section. GA: Glutaraldehyde, FA: Formaldehyde, SBF-SEM: Serial Block-Face Scanning Electron Microscopy, MIB: Microscopy Image Browser.

Journal of Cell Science • Supplementary information

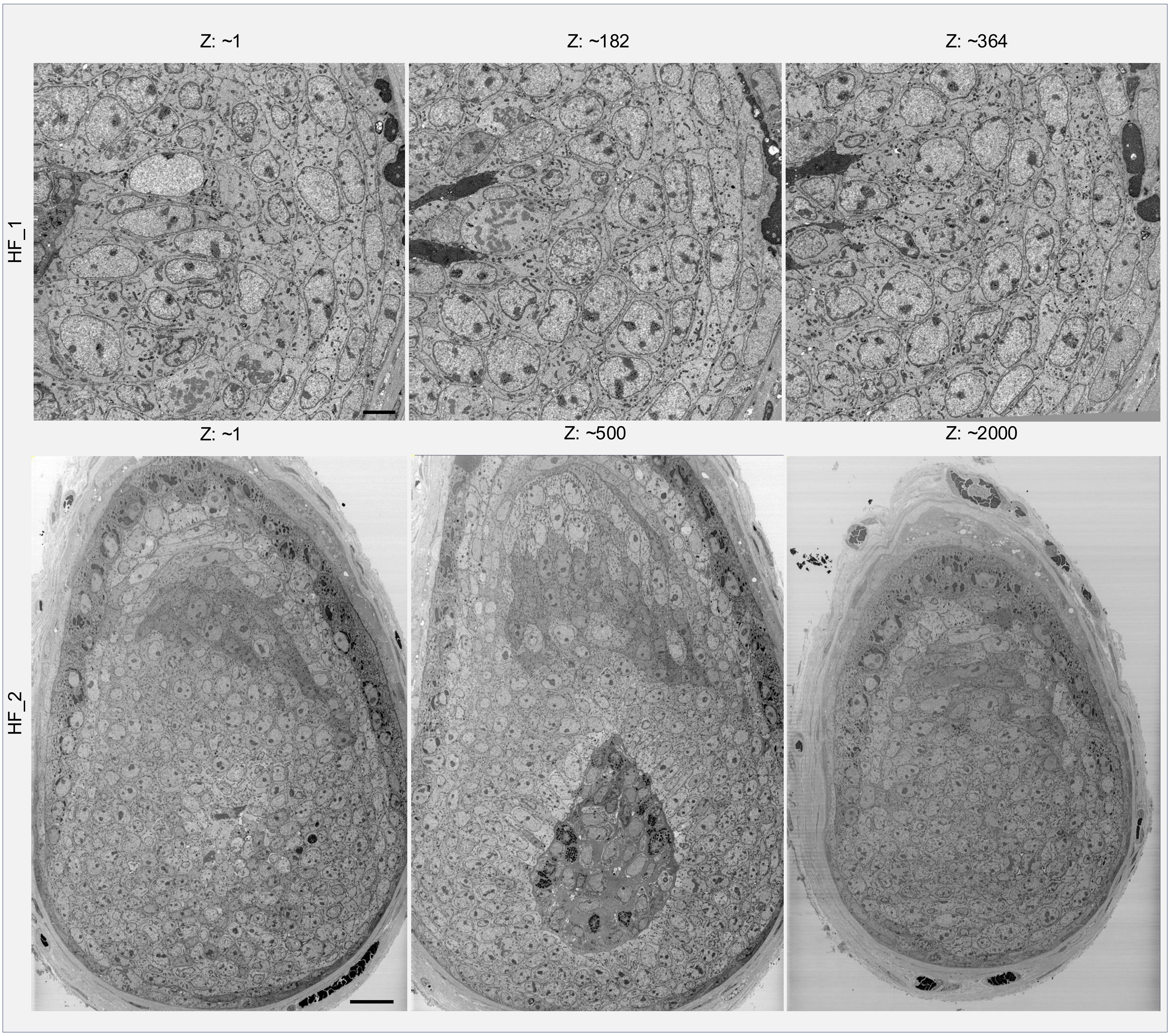

Fig. S3. H*air follicle (HF) z-stack images.* Two ovine HF Z-stack captured: HF_1 and HF_2 used in reconstructing differing dividing and non-dividing cells. Representative micrographs from selected z-slices (indicated above each panel, e.g., Z: ~1, Z: ~500) are shown. Image captured using Serial Block-Face Scanning Electron Microscopy. Scale bar to 5 (HF_1), and 20 μm (HF_2).

Journal of Cell Science • Supplementary information

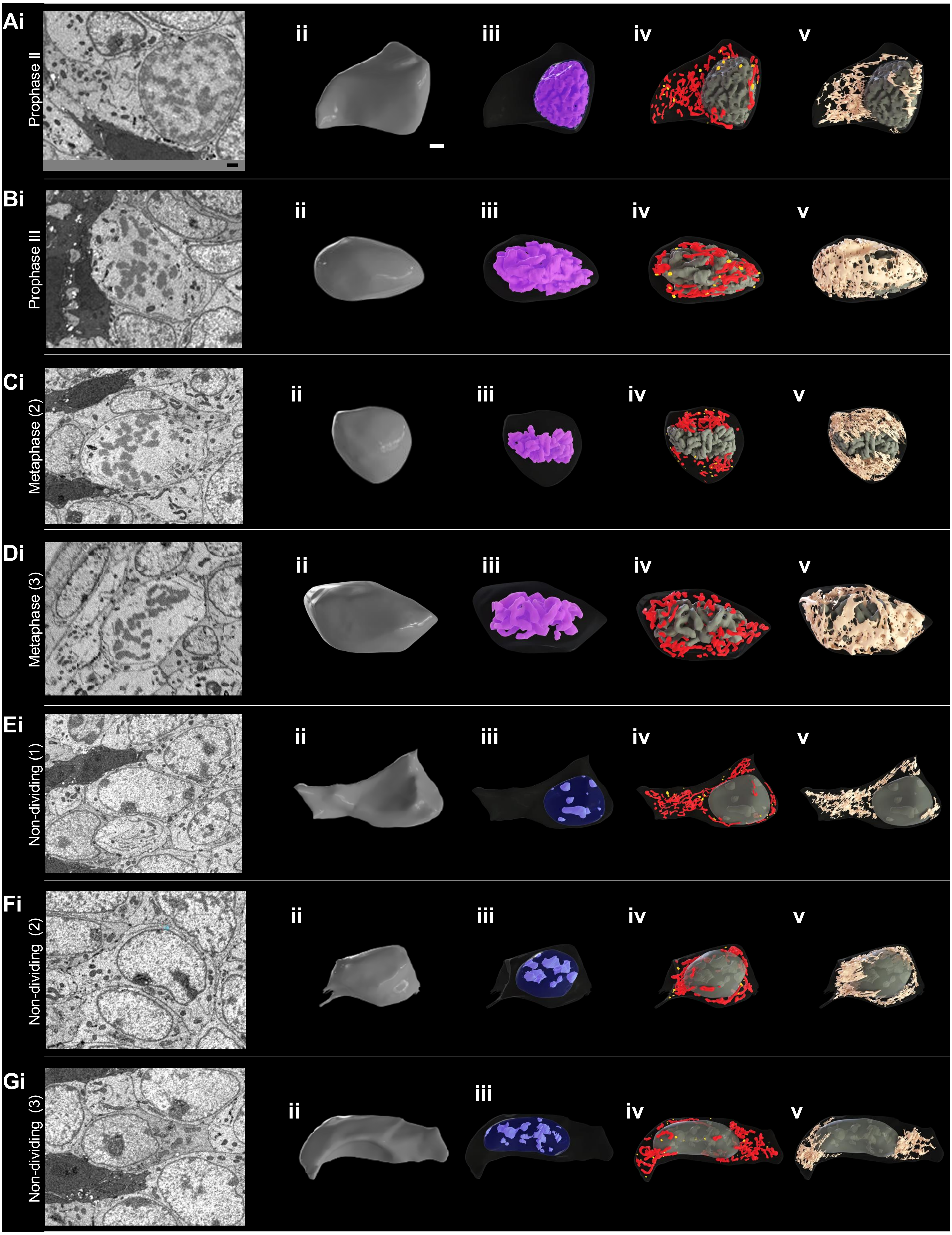

Fig. S4. ***Additional full reconstructed dividing and non-dividing cells from hair follicle.*** (**Ai-Gi**) Show representative electron micrographs of cells at various stages of cell division and non dividing states. For the same cells, columns (**ii-v**) depict 3D reconstructions of cellular and subcellular structures: (ii) cell membrane (grey), (iii) Chromosomes (purple; olive grey with sub-cellular organelles present) for most dividing cells and Nucleus for early division and non-dividing states (blue; olive grey with sub-cellular organelles present) and nucleoli (light blue). (iv) Mitochondria (red) and vesicles (yellow). (v) Endoplasmic reticulum (cream). Image were captured using an Serial Block-Face Scanning Electron Microscopy. Scale bars: 1 (Ai-Gi), and 2 µm (ii-v).

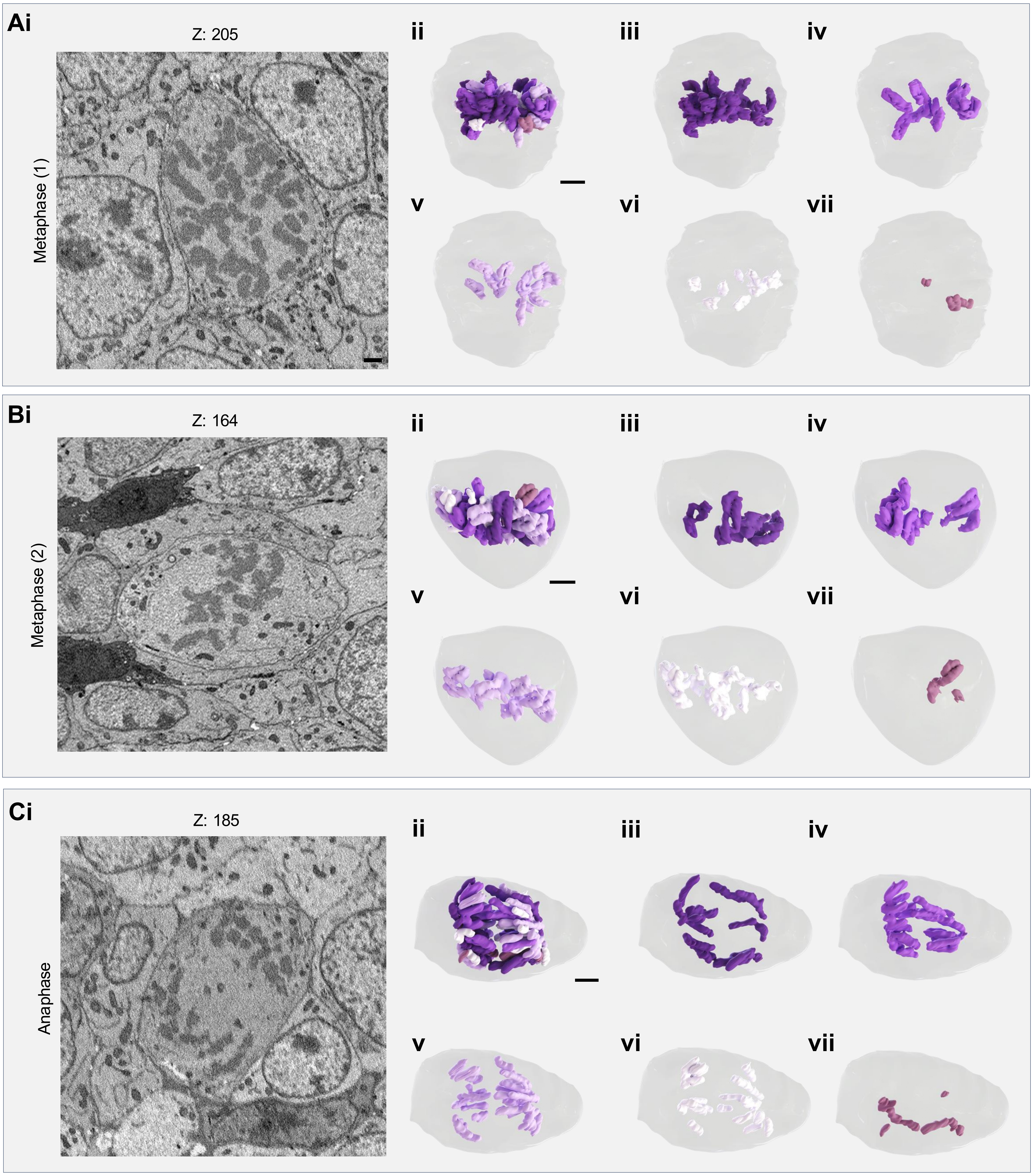

Fig. S5. ***Spatial Orientation of metaphase and anaphase assigned karyotypic groups.*** Serial Block-Face Scanning Electron Microscopy z-slice taken at the middle of the corresponding mitotic phase. Chromatids are paired and grouped into assigned volumes (see methods for more details). Chromatids are colored from purple to white in descending volume, and sex-linked chromosomes are in dark pink. (**A and B**) Reconstruction of two metaphase cells. (**C**) Reconstruction of anaphase cell. Scale bars: 1 (micrographs) and 2 µm (reconstructions).

Journal of Cell Science • Supplementary information

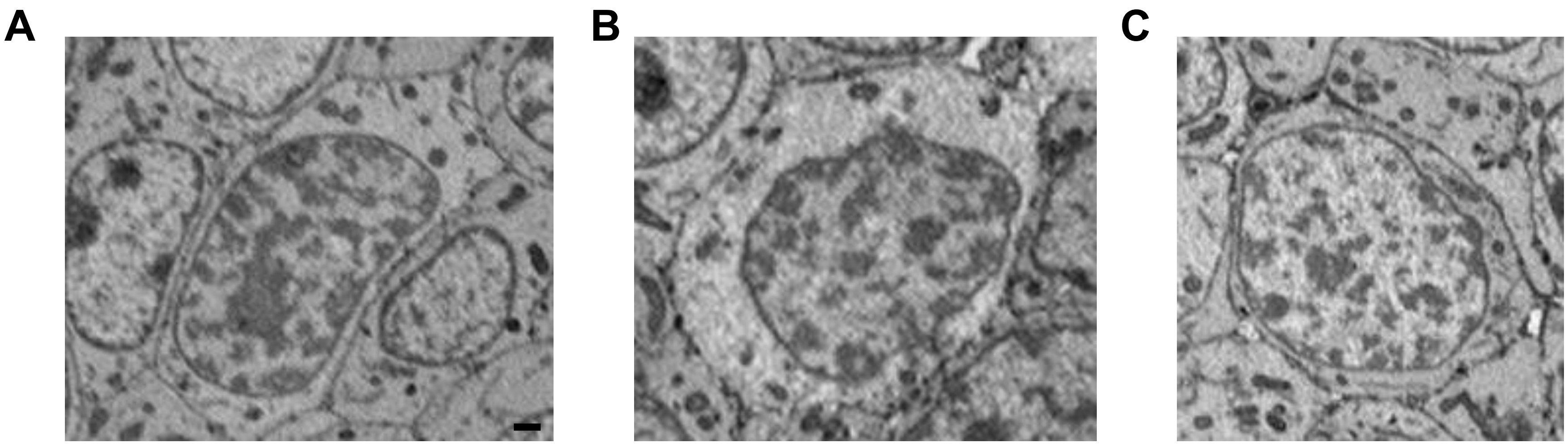

Fig. S6. ***Early stage of prophase.*** (**A-C**) Serial Block-Face Scanning Electron Microscopy micrographs of identified early stage of prophase cells. Scale to 1 um.

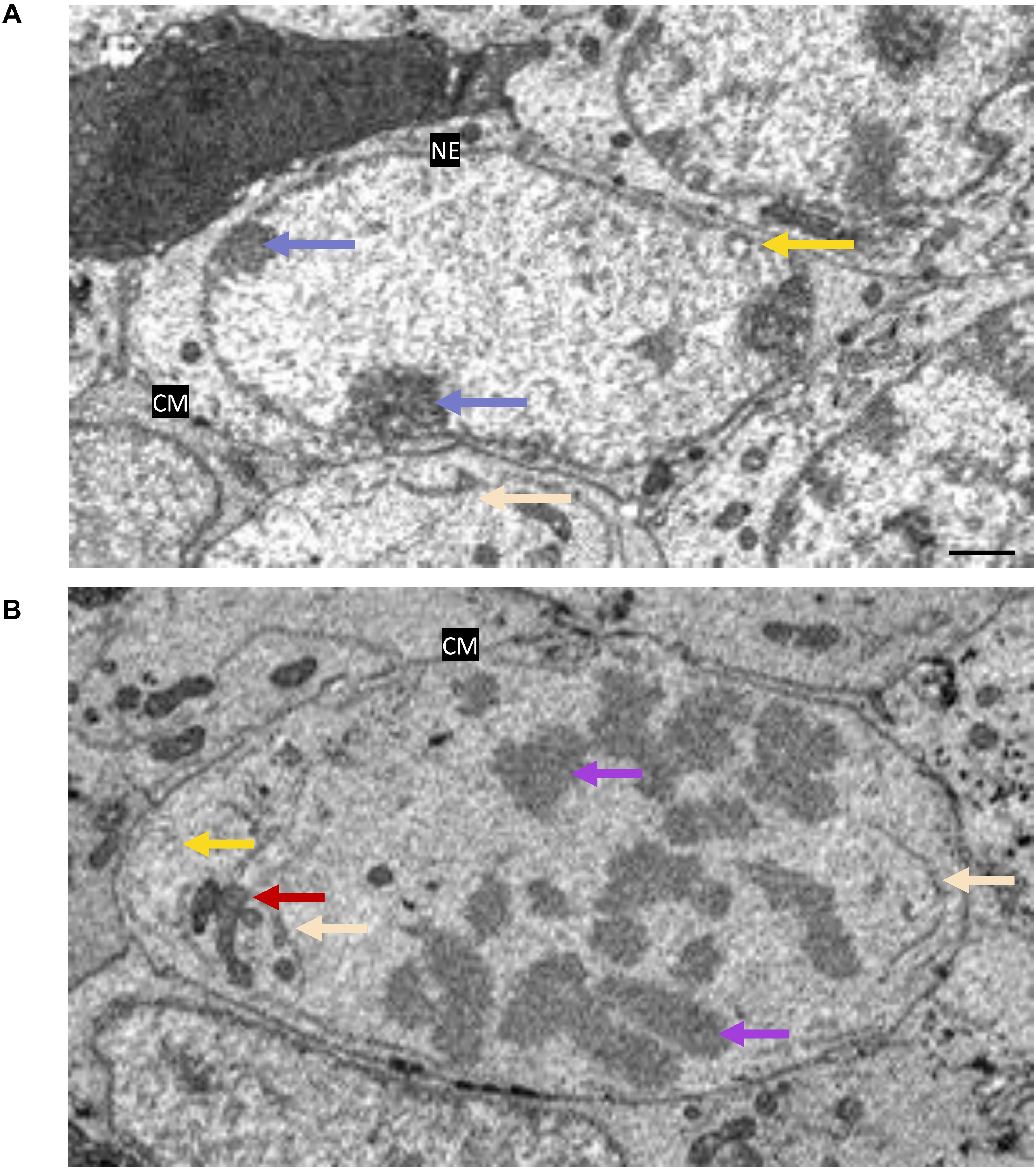

Fig. S7. ***Example of labelled cellular and sub-cellular structure in non-dividing and dividing cell.*** (**A-B**) Serial Block-Face Scanning Electron Microscopy micrographs of identified structures: Mitochondria (red), Endoplasmic Reticulum (cream), Vesicle (yellow), Nucleoli (light blue), Chromosomes (purple), NE: Nuclear Envelope, CM: Cell Membrane. Scale to 1 μm.

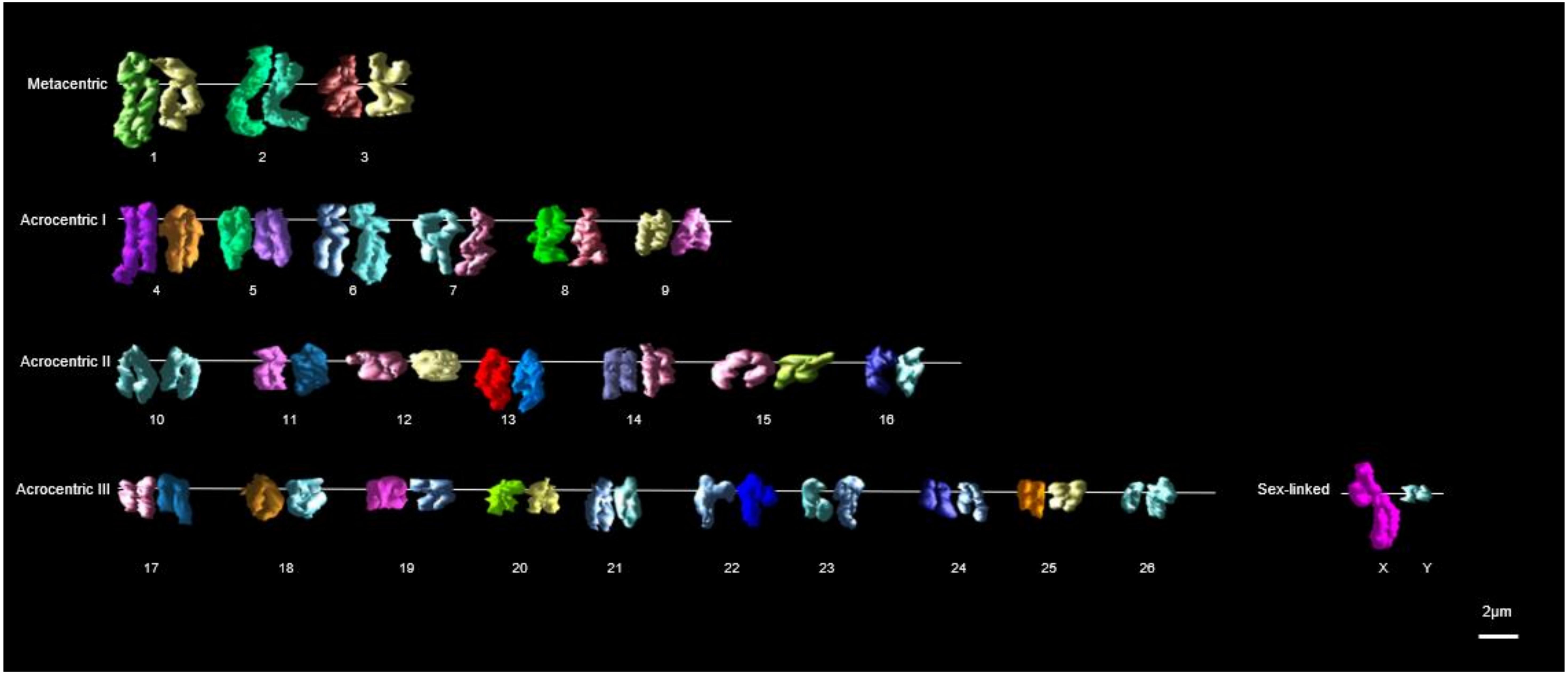

Fig. S8. ***Second karyotypic mapping of ovine chromosomes based on volume.*** (**A**) 3D reconstruction of diploid chromosomes from the second reconstructed metaphase cell, accompanied by z-stack micrograph. Line a: largest metacentric chromosomes. Lines b-d: acrocentric chromosomes grouped i-iii by descending volume. Sex-linked chromosomes presented as X, and Y. Chromosomes matched based on volume, centrosome position, spatial orientation, and distinct morphology features. Colours has no correlation to chromosome properties or order. Scale to 2 μm.

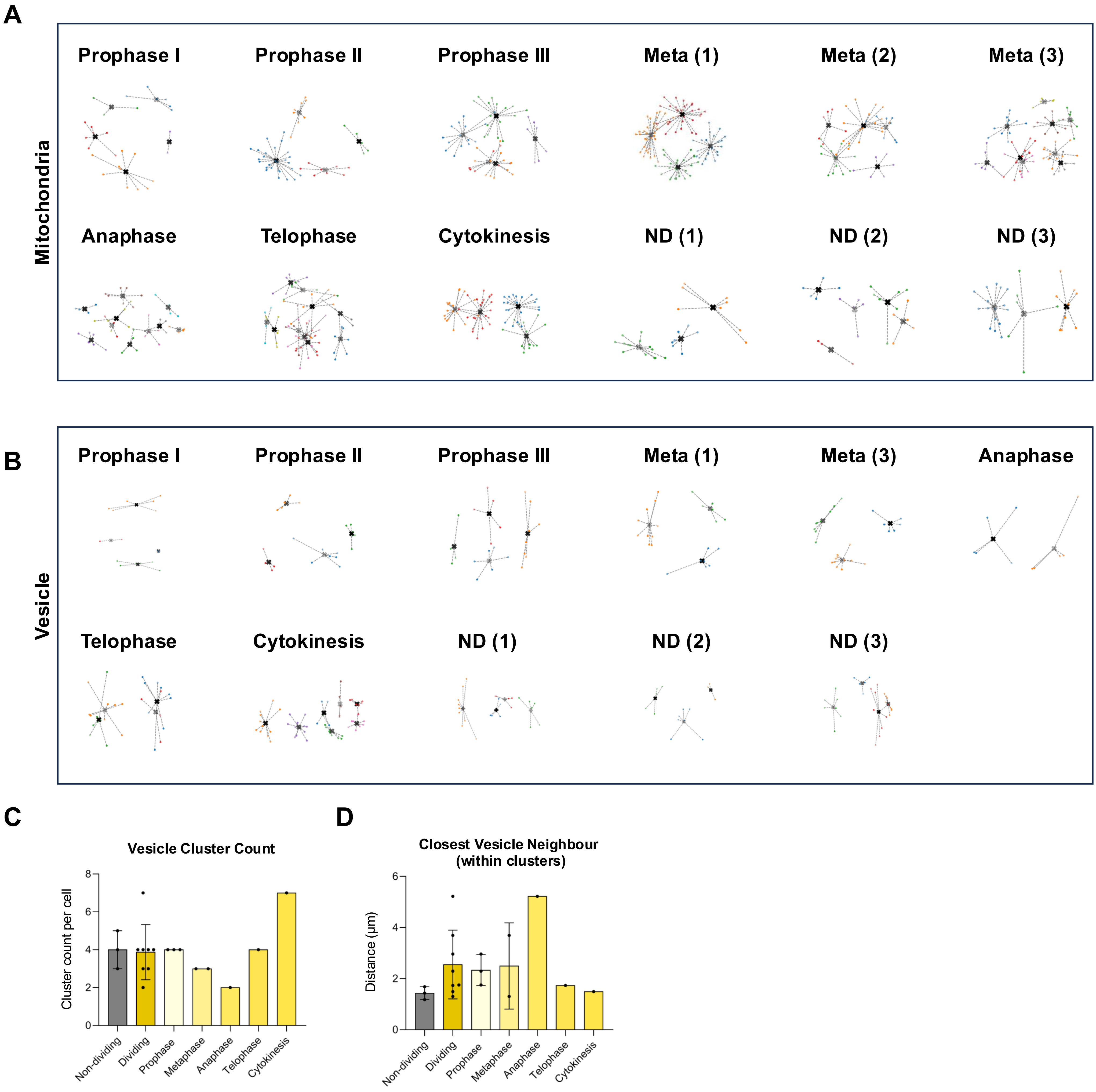

Fig. S9. C*luster analysis of mitochondria and vesicles in dividing and non-dividing cells.* Cluster analysis of mitochondria and vesicles across mitotic phases (Prophase I–III, three Metaphase cells for mitochondria and two for vesicles, Anaphase, Telophase, and Cytokinesis) and three non-dividing (ND) cells. (A) 3D mitochondrial cluster arrangement. (B) 3D vesicle cluster arrangement. (C) Vesicle cluster count per cell. (D) Closest vesicle neighbor distance (µm) within clusters. Different colours represent individual clusters, with each cluster center marked by an 'X'. For further methodological details, refer to the methods section. Data presented as means ± SD. Individual data points are shown.

Journal of Cell Science • Supplementary information

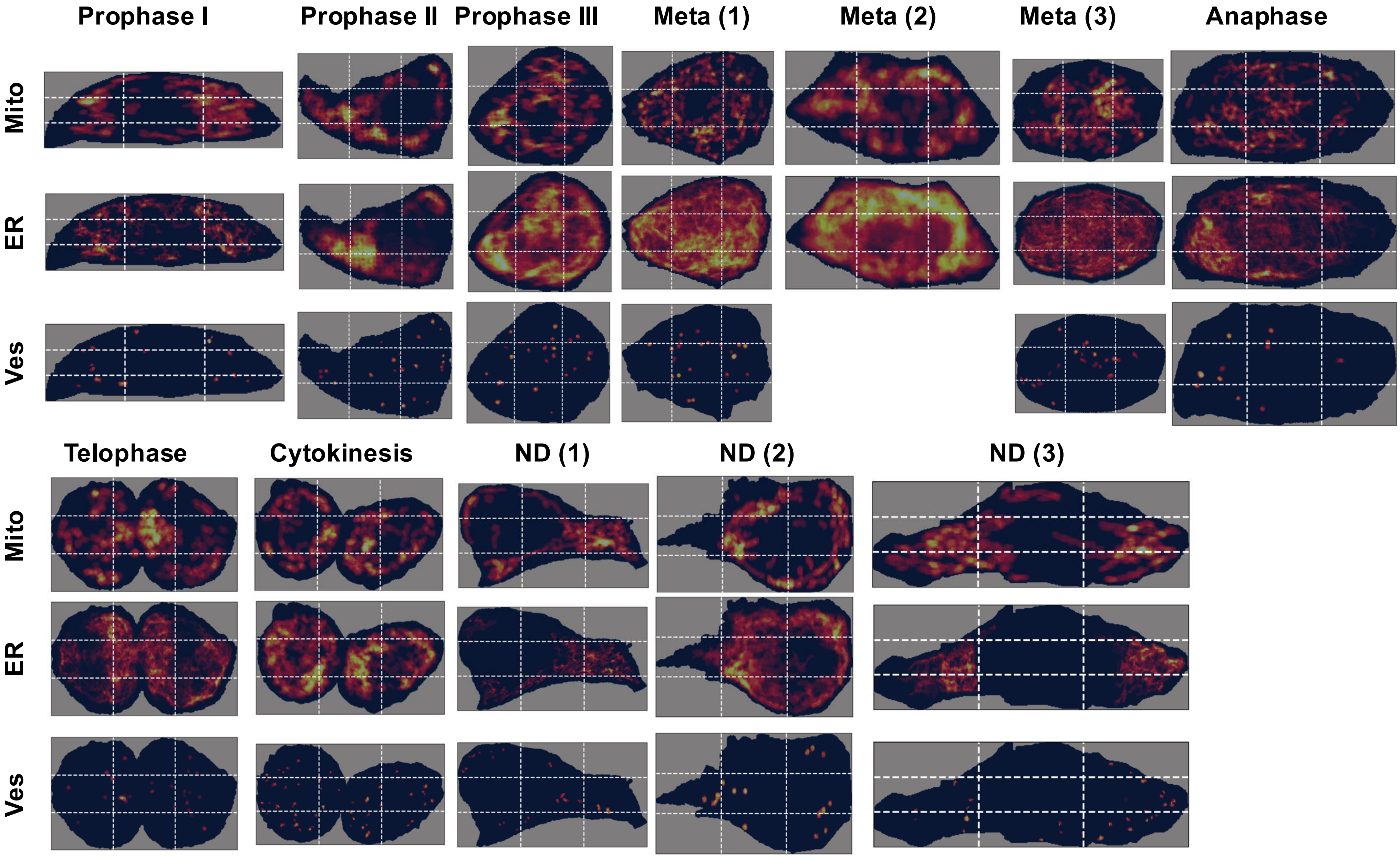

Fig. S10. ***Organelle hotspot analysis in dividing and non-dividing cells.*** Hotspot maps of mitochondria, endoplasmic reticulum (ER), and vesicles are shown for each mitotic phase (Prophase I–III, three Metaphase cells for mitochondria and two for vesicles, Anaphase, Telophase, and Cytokinesis) and three non-dividing (ND) cells overlayed on the corresponding cell membrane (dark blue). The figure is arranged in a matrix format, with mitotic phases displayed across the top and organelles listed down the side in the following order for each cell: mitochondria (top), ER (middle), and vesicles (bottom). For further methodological details, refer to the methods section.

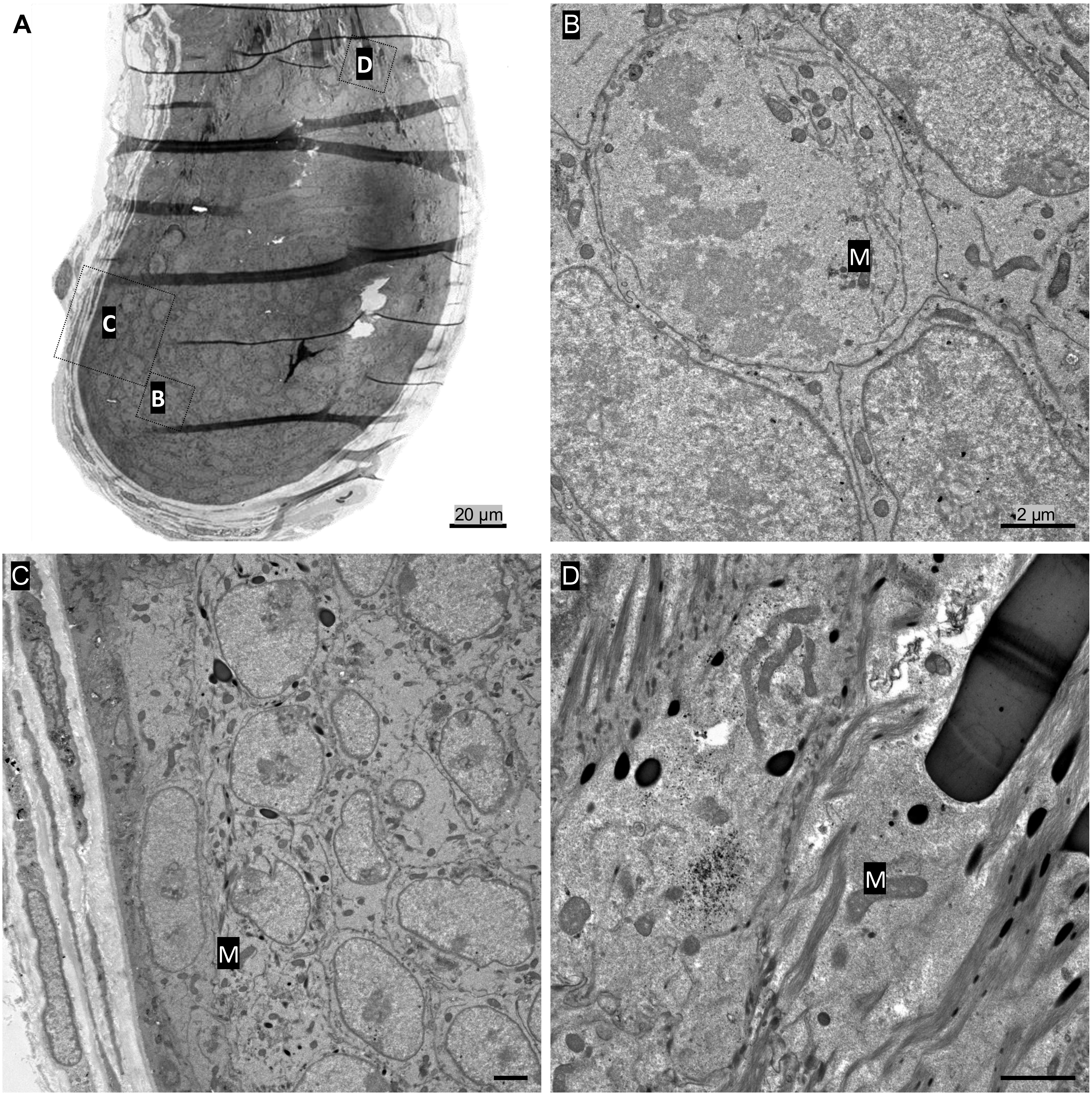

Fig. S11. ***Overview of wool hair follicles prepared for SBF-SEM.*** Samples were imaged at 100 kV using a transmission electron microscope (Morgagni 268D, Thermo Fisher Scientific, USA), with micrographs captured by a CCD camera (Tengra, Emsis, Germany). Enhanced contrast of subcellular structures, including mitochondria (M), was observed compared with conventional TEM staining methods (Velamoor et al., 2022). Images show representative regions of the lower follicle bulb (Zones A and B), including the dermal sheath and epidermal cell lineages. Scale bars: 20 µm (A) and, 2 µm (B-D).

Table S1. ***SBF-SEM image parameters.*** Serial block-face scanning electron microscopy (SBF-SEM) imaging parameters. Summary of acquisition settings for two different ovine hair follicle (HF) z-stacks (HF_1 and HF_2). Parameters include pixel time (µs), pixel size (nm), XY dimension (µm), cut thickness (nm), and number of cuts.

Parameters	HF_1	HF_2
Pixel time (µs)	4.0	4.0
Pixel size (nm)	30	30
XY dimension (µm)	60 x 60	162 x 240
Cut thickness (nm)	30	30
Number of cuts	1500	3000

Table S2. ***Number of cells analysed in-depth for each cellular component.*** This summary table presents the number of cells analysed in-depth for both non-dividing and dividing cells, distinguishing between different mitotic phases (Prophase I-III, Metaphase, Anaphase, Telophase, and Cytokinesis). The data are derived from analysis across two ovine HF samples.

Cellular components	Non-dividing	Dividing	Prophase I	Prophase II	Prophase III	Metaphase	Anaphase	Telophase	Cytokinesis
Cell membrane	5	9	1	1	1	3	1	1	1
Chromosomes	-	9	1	1	1	3	1	1	1
Nucleus	8	4	1	1	-	-	-	1	1
Mitochondria	5	9	1	1	1	3	1	1	1
Vesicles	3	8	1	1	1	2	1	1	1
Endoplasmic reticulum	3	9	1	1	1	3	1	1	1

Table S3. ***Statistical significance table for Karyotypic group surface area (µm²), and Surface area-to-volume ratio of metaphase cells.*** This summary table represents the statistical significance for Figure 3C and E. Comparing the karyotypic group of metaphase cells between each other for differences in surface area and surface area/ volume.

	Karyotypic group	Metacentric	Acrocentric I	Acrocentric II	Acrocentric III	X-linked	Y-linked
Surface Area	Metacentric	-	****	****	****	ns	****
	Acrocentric I	****	-	****	****	ns	***
	Acrocentric II	****	****	-	****	*	*
	Acrocentric III	****	****	****	-	*	*
	X-linked	ns	ns	*	*	-	**
	Y-linked	****	***	*	*	**	-
Surface Area/ Volume	Metacentric	-	ns	ns	****	ns	ns
	Acrocentric I	ns	-	ns	****	ns	ns
	Acrocentric II	ns	ns	-	***	ns	ns
	Acrocentric III	***	****	***	-	**	ns
	X-linked	ns	ns	ns	**	-	ns
	Y-linked	ns	ns	ns	ns	ns	-

Data presented as means ± SD. *P ≤ 0.05, **P ≤ 0.01, ***P ≤ 0.001, ****P ≤ 0.0001, and ns: not significant

Table S4. ***Summary of endoplasmic reticulum (ER) volume composition analysisni mitotically and non-dividing cells.*** Long fragments (>1 µm³), medium fragments (0.1 – 1 µm³), and short fragments (<0.01 µm³).

ER	Non-Dividing			Prophase I			Prophase II			Prophase II			Metaphase			Anaphase			Telophase			Cytokinesis		
	(v)	(n)	(%)	(v)	(n)	(%)	(v)	(n)	(%)	(v)	(n)	(%)	(v)	(n)	(%)	(v)	(n)	(%)	(v)	(n)	(%)	(v)	(n)	(%)
Long	14.1	1.7	97	18.8	6	75	58.4	2	98	134.0	1	100	122.8	1	100	53.0	1	92	38.3	4	92	30.1	7	73
Medium	0.4	1	3	6.0	14	24	1.2	3	2	-	-	-	-	-	-	2.9	10	5	1.8	8	4	9.5	25	23
Short	-	-	-	0.1	2	1	-	-	-	-	-	-	-	-	-	1.7	45	3	1.5	37	4	1.5	31	4

Number of cells analysed = Non-Dividing and Metaphase (n=3), Prophase – prophase III and anaphase – cytokinesis (n=1).
Non-dividing and metaphase shown as average.
(v) = Total volume of ER fragment.
(n) = Total number of ER fragments based on volume
(%) = Volume of ER fragments as a percentage of total ER volume within the cell.
ER fragments are categorised by volume as follows: long (>1 µm³), medium 0.1 – 0.1 µm³), and short <0.1 µm³).
